# Amelioration Effects of *Pseudomonas fluorescens* on Acidified Soil and Subsequent Promotion of Maize Growth

**DOI:** 10.3390/plants15172679

**Published:** 2026-08-31

**Authors:** Tingyi Wang, Ze Yu, Meilan Chen, Lansheng Deng

**Affiliations:** College of Natural Resources and Environment, South China Agricultural University, Guangzhou 510640, China; wtyy1214@stu.scau.edu.cn (T.W.); yze@stu.scau.edu.cn (Z.Y.); 20252020001@stu.scau.edu.cn (M.C.)

**Keywords:** *Pseudomonas fluorescens*, soil acidification, organic matter, maize, rhizosphere microorganisms

## Abstract

Soil acidification severely restricts agricultural productivity in red soil regions, and microbial remediation represents an eco-friendly restoration strategy. A pot experiment was conducted to investigate the dose-dependent effects of *Pseudomonas fluorescens* on acidified latosol, maize seedling growth, and rhizosphere bacterial communities. Five inoculation rates were applied (5.5 × 10^9^–3.3 × 10^10^ CFU pot^−1^), together with a non-inoculated control. *Pseudomonas fluorescens* inoculation significantly increased soil pH and base cation contents, reduced exchangeable and hydrolytic acidity, and enhanced soil acid–base buffering capacity. Low to moderate inoculation rates promoted maize photosynthesis, root development, and nitrogen, phosphorus, and potassium accumulation, whereas the highest rate (3.3 × 10^10^ CFU pot^−1^) significantly inhibited seedling growth. Bacterial sequencing showed that inoculation reshaped rhizosphere community structure and enriched taxa, including Proteobacteria, Bacillota, *Bacillus*, and *Pseudomonas*. Overall, inoculation rates of 1.1 × 10^10^ and 2.2 × 10^10^ CFU pot^−1^ showed the most favorable responses across soil amelioration and maize growth-related traits, suggesting a suitable application range for acidified soil remediation. These results provide preliminary evidence that appropriate application of *Pseudomonas fluorescens* may contribute to the biological amelioration of acidified soils, although its field-scale effectiveness requires further validation.

## 1. Introduction

Soil acidification has become an increasingly important constraint on agricultural productivity, particularly in intensively managed red-soil regions [1,2]. Progressive acidification increases soil H^+^ activity and the solubility of phytotoxic Al^3+^, alters the balance of exchangeable base cations, and can reduce the availability of essential nutrients, thereby impairing root development, nutrient acquisition, plant physiological performance, and ultimately crop productivity [3,4,5]. Long-term and excessive application of mineral fertilizers, especially nitrogen (N) fertilizers, may further accelerate soil acidification, nutrient leaching, and deterioration of soil physicochemical properties, creating a negative feedback loop between declining soil quality and increasing fertilizer inputs. Conventional amelioration practices such as liming can rapidly increase soil pH, but their effectiveness depends strongly on application rate and soil conditions, and excessive amendment may induce nutrient imbalance or over-alkalization. Therefore, developing environmentally sustainable strategies that alleviate soil acidity while simultaneously improving nutrient availability, rhizosphere functioning, and crop performance is important for the sustainable management of acidified farmland.

Plant growth-promoting rhizobacteria provide a potential biological approach for improving degraded soil environments. Among them, *Pseudomonas fluorescens* is widely distributed in agricultural soils and plant rhizospheres and exhibits strong rhizosphere colonization capacity and metabolic adaptability [6,7,8,9,10]. Previous studies have demonstrated that *Pseudomonas fluorescens* can promote plant growth through multiple mechanisms, including regulation of nutrient availability, production of plant growth-promoting metabolites, competition with soil-borne pathogens, and modulation of rhizosphere microbial communities [11,12,13,14]. Its adaptation and functional activities in the rhizosphere are partly regulated by signaling systems such as the GacS/GacA two-component system, which coordinates several traits related to secondary metabolism, stress adaptation, and rhizosphere competence [15,16,17,18]. In acidified soils, *Pseudomonas fluorescens* has also been proposed to contribute to soil amelioration through both physicochemical and biological processes. Previous studies suggest that microbial cell-surface functional groups may bind H^+^, while microbial metabolites such as organic acids and extracellular polymeric substances may facilitate mineral weathering and the release of base cations [19,20,21]. These processes may contribute to reductions in exchangeable acidity and changes in soil buffering capacity. In addition, *Pseudomonas fluorescens* inoculation has been reported to alter rhizosphere bacterial community structure and favor plant growth-promoting taxa, potentially improving nutrient cycling and rhizosphere functioning [13,19,22].

Despite the broad application potential of *Pseudomonas fluorescens*, most previous studies have focused on its biocontrol activity, plant growth-promoting functions, or tolerance to individual environmental stresses [23,24,25,26], whereas its integrated effects on strongly acidified agricultural soils remain less well characterized [27]. In particular, the response of acidified soil to increasing inoculation rates may not be linear. Moderate inoculation may alleviate soil acidity and improve rhizosphere conditions, whereas excessive application may substantially alter soil ionic strength, nutrient balance, or microbial interactions and thereby reduce the beneficial effects on plants [28]. Moreover, relatively few studies have simultaneously evaluated changes in soil acidity-related properties, plant physiological and root responses, nutrient accumulation, and rhizosphere bacterial community structure across a broad inoculation gradient [29]. Consequently, it remains unclear whether increasing *Pseudomonas fluorescens* application consistently improves acidified soils or whether an optimal application range exists beyond which the beneficial effects decline. Clarifying this dose-dependent response is important for understanding the ecological effects of microbial inoculation and for establishing appropriate application strategies for acidified farmland.

The main objective of this study was to determine how different inoculation concentrations of *Pseudomonas fluorescens* affect acidified soil properties, maize seedling performance, and rhizosphere bacterial communities. Unlike previous studies that mainly emphasized the beneficial effects of *Pseudomonas fluorescens* under single or limited application levels, this study focused on a broad inoculation gradient to resolve potential non-linear dose responses and identify an appropriate application range. To achieve this, we conducted a pot experiment using acidified latosol and established five inoculation rates of a commercial *Pseudomonas fluorescens* formulation, together with a non-inoculated control. We quantified soil acidity-related properties, exchangeable base cations, nutrient status, acid–base buffering capacity, maize growth and photosynthetic traits, root morphology, plant N, phosphorus (P), and potassium (K) accumulation, and rhizosphere bacterial diversity and community composition using 16S rRNA gene sequencing. Based on the potential non-linear response to increasing inoculation rates, we hypothesized that: (1) moderate inoculation rates would produce the most favorable responses in soil acidity alleviation, soil nutrient conditions, and maize growth, whereas excessive inoculation would weaken or reverse these beneficial effects; and (2) moderate inoculation rates would induce more favorable shifts in rhizosphere bacterial community composition and enhance the relative abundance of plant growth-promoting taxa, whereas excessive inoculation would cause divergent changes in community structure.

## 2. Materials and Methods

### 2.1. Experimental Materials

Tested microbial inoculant: The microbial inoculant used was a wettable powder of *Pseudomonas fluorescens*, purchased from Shandong Tainuo Pharmaceutical Co., Ltd, Weifang City, Shandong Province, China. The strain adopted for inoculant fermentation is *F113*, a classic international model strain with rhizosphere growth-promoting and biocontrol functions. Its complete genome sequence has been deposited in GenBank under the nucleotide accession number CP003150. All physicochemical indicators were determined in accordance with the national standard GB 20287-2006 for agricultural microbial inoculants: viable count 5.5 × 10^8^ CFU g^−1^, moisture content 2.5%, fineness (passing through a 74 μm sieve) 99%, suspension rate 82%, pH 7.7, electrical conductivity (EC) 3.2 mS cm^−1^, total carbon (TC) 85.1 g kg^−1^, total nitrogen (TN) 11.8 g kg^−1^, and total phosphorus (TP) 3.5 g kg^−1^.

Test fertilizers: Urea [CO(NH_2_)_2_], potassium sulfate (K_2_SO_4_), and ammonium dihydrogen phosphate (NH_4_H_2_PO_4_).

Test maize cultivar: Zhengdan 957.

Test soil: The test soil was latosol collected from Zhanjiang, Guangdong Province. Its basic physicochemical properties were measured following the methods described in Section 2.4. The baseline properties were as follows: pH 4.52, EC 180.33 μS cm^−1^, soil organic matter 7.52 g kg^−1^, available phosphorus 2.36 mg kg^−1^, TN 0.48 g kg^−1^, available potassium 34.81 mg kg^−1^, exchangeable calcium 0.03 g kg^−1^, exchangeable magnesium 0.34 g kg^−1^, exchangeable sodium 0.11 g kg^−1^, exchangeable hydrogen 1.28 cmol(c) kg^−1^, and exchangeable aluminum 9.42 cmol(c) kg^−1^.

### 2.2. Experimental Design

The preliminary experiment was carried out from April to May 2025, and the formal experiment was conducted from September to October 2025. Both trials were performed at the experimental base of the Crop Fertilization Laboratory, South China Agricultural University (113.354° E, 23.158° N). Stable environmental conditions were maintained throughout the experimental period, with a maximum temperature of 32 °C, a minimum temperature of 19 °C, an average air temperature of 26.3 °C, and an average relative air humidity of 76.1%.

Maize seeds were surface-sterilized and germinated in seedling trays under constant temperature and light conditions: a temperature of 25 °C, relative humidity of 90%, and photosynthetic photon flux density of 250 μmol m^−2^ s^−1^. When maize seedlings reached the two-leaf-one-heart stage, robust, undamaged seedlings with uniform growth vigor were selected for transplanting. Uniform cylindrical plastic pots were used in the experiment, with an upper inner diameter of 19 cm, a bottom diameter of 15.5 cm, and a height of 20 cm. Matching water-retaining trays with a diameter of 17.9 cm were provided for each pot, and one maize seedling was planted per pot. The experiment adopted a completely randomized design, and all pots were cultured and managed under consistent environmental conditions at the experimental base.

Prior to pot filling, the test soil was sieved through a 2 mm screen, with 2.0 kg of soil quantitatively filled into each pot. The soil field capacity (FC) was pre-determined via the core cutting method. During cultivation, soil moisture content was monitored at 08:00 and 20:00 every day using a soil moisture sensor (Delta-T ML2x). The supplementary water volume was calculated according to the formula: W = (0.8FC − t) × VS, where W represents supplementary water volume; FC is soil field capacity; t denotes real-time soil moisture content; and VS refers to the total soil volume per pot. Water was added slowly and evenly to maintain soil moisture at 80% of field capacity, ensuring consistent soil water conditions across all treatments and eliminating water stress and inter-pot variations.

Six treatments were established in this pot experiment, including a blank control CK (no inoculation of *Pseudomonas fluorescens*, 0 CFU pot^−1^ and five gradient inoculation treatments (T1–T5). The strain inoculation amounts for T1, T2, T3, T4 and T5 were 5.5 × 10^9^, 8.25 × 10^9^, 1.1 × 10^10^, 2.2 × 10^10^ and 3.3 × 10^10^ CFU pot^−1^, corresponding to commercial inoculant application rates of 10, 15, 20, 40 and 60 g pot^−1^, respectively. A total of six treatments were established, with six pots prepared for each treatment, resulting in 36 pots in total. Each pot received the treatment independently and contained one maize plant. Therefore, the pot was considered the experimental unit. Four independent pots per treatment were used for measurements and statistical analysis (*n* = 4), while the remaining two pots were maintained as backup replicates. The inoculation concentration gradients for the formal experiment were screened from a preliminary gradient trial. Multiple inoculant addition levels were set in the preliminary trial. Using maize seedling growth performance and soil physicochemical properties as evaluation indices, the suitable application concentration range of the inoculant was identified, and the target viable-cell concentration for each treatment in the formal experiment was determined accordingly. Cultivation management and fertilization conditions were identical across all treatments. Urea (0.17 g), potassium sulfate (0.2 g) and ammonium dihydrogen phosphate (0.32 g) were applied uniformly to each pot. The only variable among treatments was the application concentration of *Pseudomonas fluorescens*. During experimental preparation, the inoculant mass required per pot was converted and accurately weighed according to the measured viable-cell count of the test inoculant and the preset target viable-cell concentration in soil for each treatment. The weighed inoculant was thoroughly mixed with the test soil to ensure homogeneous distribution of bacterial cells within the soil, followed by pot filling.

### 2.3. Sample Collection

To eliminate data fluctuations caused by individual differences in plant growth, four plants with uniform and consistent growth performance were selected to measure all indicators during each sampling, and the remaining two pots were used as spare parallel replicates.

Standard pretreatment procedure for plant samples includes fixation at 105 °C. The core function of this step is to rapidly inactivate various oxidases and hydrolases in plant tissues under high temperature, inhibit the decomposition and transformation of chlorophyll, soluble sugars, amino acids, nutrients and other metabolites, maximally retain the original nutrients and physiological compounds inside plants, and prevent component loss and biased test results during drying. Detailed operational steps are as follows: Thoroughly cleaned roots, stems and leaves were placed into clean aluminum boxes, incubated in a forced-air drying oven at 105 °C for 20 min for fixation. Then, the oven temperature was adjusted to 65 °C and the samples were continuously dried until they reached a constant weight to weigh the dry biomass of each plant organ.

Plant morphological and chlorophyll indices: Plant height was measured with a tape measure; stem diameter was determined 1 cm above the soil surface using a vernier caliper; the relative SPAD chlorophyll values of functional leaves were detected via an SPAD-502 chlorophyll meter. Root images were scanned using the RHIZO 2008 root analysis system, and the supporting software was applied to calculate total root length, total root volume and total root surface area. A LI-6000 photosynthesis system was used to uniformly measure the net photosynthetic rate and transpiration rate of functional leaves from 9:00 to 11:00 a.m. before sampling.

Plant nutrient indices: Dried root, stem and leaf samples were ground and sieved through a 0.15 mm mesh for standby use. TN in plants was determined by the Kjeldahl digestion-semi-micro distillation titration method. After dilution of the digestion solution, TP was quantified by the vanadium–molybdenum yellow colorimetric method, and TK was measured using flame photometry.

Collection and pretreatment of soil samples: After removing the aboveground plant parts, the soil was entirely separated from each pot. The plants were gently lifted by the root crown to shed bulk soil naturally. Sterile tweezers were used to carefully strip the 0–2 mm soil layer tightly attached to root surfaces, which was collected as rhizosphere soil. Rhizosphere soil samples were sealed, transported back to the laboratory within 6 h, and immediately stored at −80 °C for bacterial 16S rRNA gene sequencing. Bulk soil was sampled using the quartering method, air-dried naturally, and sequentially sieved through 1 mm (18-mesh), 0.25 mm (60-mesh) and 0.15 mm (100-mesh) sieves for the analysis of soil physicochemical properties.

### 2.4. Sample Determination

#### 2.4.1. Determination of Conventional Soil Chemical Indices

Soil nutrient and acidification-related indices were determined following the standard methods described in Soil Agricultural-Chemical Analysis (3rd edition) by Bao Shidan [30]. Soil pH and EC were measured using a pH meter and conductivity meter after extraction with soil-water mass ratios of 2.5:1 and 5:1, respectively. SOM was determined by the external-heating potassium dichromate oxidation method. Soil AP was extracted by the Olsen method and quantified via molybdenum–antimony–ascorbic acid colorimetry. Soil TN was measured using the Kjeldahl digestion-semi-micro distillation method. The soil C/N was calculated from soil organic C and TN contents.

#### 2.4.2. Determination of Soil Acidification Characteristic Indices

Soil exchangeable potassium and exchangeable sodium were extracted with a 1 mol/L ammonium acetate solution and quantified by flame photometry. Soil exchangeable calcium and magnesium were determined in accordance with the standards NY/T 1121.13-2006 and NY/T 1121.14-2006 respectively. Samples were extracted with 1 mol/L ammonium acetate solution and then analyzed by atomic absorption spectrophotometry. Soil exchangeable acidity (exchangeable hydrogen and exchangeable aluminum) was tested following the standard HJ 649-2013, “Soil—Determination of exchangeable acidity—Potassium chloride extraction-titration method.” Briefly, soil samples were extracted with a 1 mol/L potassium chloride solution. The total exchangeable acidity of the extract was determined by neutralization titration. Another aliquot of the extract was treated with potassium fluoride to complex aluminum ions prior to titration, and the contents of exchangeable hydrogen and exchangeable aluminum were calculated separately. Soil hydrolytic acidity was measured by hydrolysis extraction with a 1 mol/L sodium acetate solution (pH 8.3) combined with neutralization titration. Soil acid–base buffering capacity was determined via acid–base titration. Different concentrations of acid were added to soil suspensions. After equilibrium, the changes in pH were recorded to plot the buffering curve, and the buffering capacity was calculated accordingly.

#### 2.4.3. Microbial and Bioinformatic Methods

##### DNA Extraction from Samples

Total genomic DNA of microbial communities was extracted using the E.Z.N.A.^®^ Soil DNA Kit (Omega Bio-Tek, Norcross, GA, USA) following the manufacturer’s instructions. The quality of extracted genomic DNA was verified by 1% agarose gel electrophoresis. DNA concentration and purity were determined with a Nanodrop 2000 spectrophotometer (Thermo Scientific, Waltham, MA, USA).

##### PCR Amplification and Sequencing Library Construction

Using the extracted DNA as a template, the V3-V4 hypervariable regions of the 16S rRNA gene were amplified via PCR with the barcoded forward primer 338F (5′-ACTCCTACGGGAGGCAGCAG-3′) and reverse primer 806R (5′-GGACTACHVGGGTWTCTAAT-3′). The PCR reaction system (total volume: 20 μL) was composed of 4 μL 5 × TransStart FastPfu Buffer, 2 μL 2.5 mM dNTPs, 0.8 μL forward primer (5 μM), 0.8 μL reverse primer (5 μM), 0.4 μL TransStart FastPfu DNA Polymerase, and 10 ng template DNA. The amplification program was set as follows: initial denaturation at 95 °C for 3 min; 27 cycles of denaturation at 95 °C for 30 s, annealing at 55 °C for 30 s, and extension at 72 °C for 30 s; followed by a final extension at 72 °C for 10 min. The products were stored at 4 °C. PCR amplification was performed on an ABI GeneAmp^®^ 9700 (Applied Biosystems, Foster City, CA, USA) thermal cycler. PCR products were recovered using 2% agarose gel and purified with a DNA Gel Extraction & PCR Clean-Up Kit (Yuhua, Nanjing, China). The purified products were quantified using Qubit 4.0 (Thermo Fisher Scientific, USA).

Subsequently, sequencing libraries were constructed from the purified PCR products using the NEXTFLEX Rapid DNA-Seq Kit, which included four steps: (1) adapter ligation; (2) removal of self-ligated adapters via magnetic bead screening; (3) enrichment of library templates by PCR amplification; and (4) recovery of final libraries using magnetic beads. High-throughput sequencing was carried out on the Illumina Nextseq2000 platform at Majorbio Bio-Pharm Technology Co., Ltd. (Shanghai, China). All raw microbial sequencing data from this study have been deposited in the Sequence Read Archive (SRA) database of NCBI. The BioProject accession number is PRJNA1474281, and the SRA submission number is SUB16228186.

### 2.5. Data Processing

All statistical analyses of soil physicochemical properties in this study were performed using GraphPad Prism 10.4.1. Prior to one-way analysis of variance (ANOVA), the Shapiro-Wilk test was used to verify data normality, and Bartlett’s test was applied to assess homogeneity of variances among groups. All datasets met the prerequisites for parametric tests, including normal distribution and homoscedasticity. Accordingly, one-way ANOVA combined with Tukey’s multiple comparison test was adopted to analyze differences between treatments, with the significance level set at *p* < 0.05. For one-way ANOVA, the coefficient of determination (r^2^) reported by GraphPad Prism was used to describe the proportion of total variance explained by the treatment factor and was presented as a measure of effect magnitude. Exact *p*-values were reported wherever available; for Tukey’s multiple-comparison tests, adjusted *p*-values were reported as provided by GraphPad Prism, and when the software output was limited to *p* < 0.0001, this threshold notation was retained. Raw data were initially processed with Excel 2019, and all figures and graphs were generated using GraphPad Prism 10.4.1.

Raw paired-end reads were quality-filtered with fastp v0.19.6 and merged using FLASH v1.2.1. Low-quality bases (Q < 20) at read tails were trimmed with a 50 bp sliding window (average Q < 20); reads shorter than 50 bp or containing ambiguous bases (N) were removed. Paired-end reads were merged with a minimum overlap of 10 bp and a maximum mismatch rate of 0.2. Samples were demultiplexed by barcode (0 mismatches) and primers (≤2 mismatches), with sequences oriented correctly. Cleaned sequences were clustered into OTUs (97% similarity) and chimeras removed using UPARSE v7.1. All samples were rarefied to 20,000 sequences (average Good’s coverage = 99.09%). Taxonomic classification was performed with RDP Classifier v2.11 against the Silva v138 database (confidence threshold = 70%).

All data analyses were performed on the Majorbio Cloud Platform (https://cloud.majorbio.com). The Chao1, Shannon and other alpha diversity indices were calculated using mothur, and intergroup differences were analyzed via the Wilcoxon rank-sum test. Principal component analysis (PCA) was performed based on bacterial community abundance data to visualize differences in overall community composition among treatments. Principal coordinate analysis (PCoA) based on Bray–Curtis dissimilarity was further used to assess differences in bacterial community structure among samples. Analysis of similarities (ANOSIM) was applied to test whether between-treatment differences were greater than within-treatment variation. Linear discriminant analysis effect size (LEfSe) (LDA > 2, *p* < 0.05) was adopted to identify differentially abundant bacterial taxa from phylum to genus levels. Distance-based redundancy analysis (db-RDA) was conducted to explore the effects of soil physicochemical properties on bacterial communities. Linear regression analysis was used to evaluate the relationships between key soil factors and alpha diversity indices. Species for co-occurrence network analysis were selected based on Spearman’s correlation (|r| > 0.6, *p* < 0.05).

## 3. Results

### 3.1. Effects of Pseudomonas fluorescens on Soil Physicochemical Properties and Nutrients

**Figure 1:**
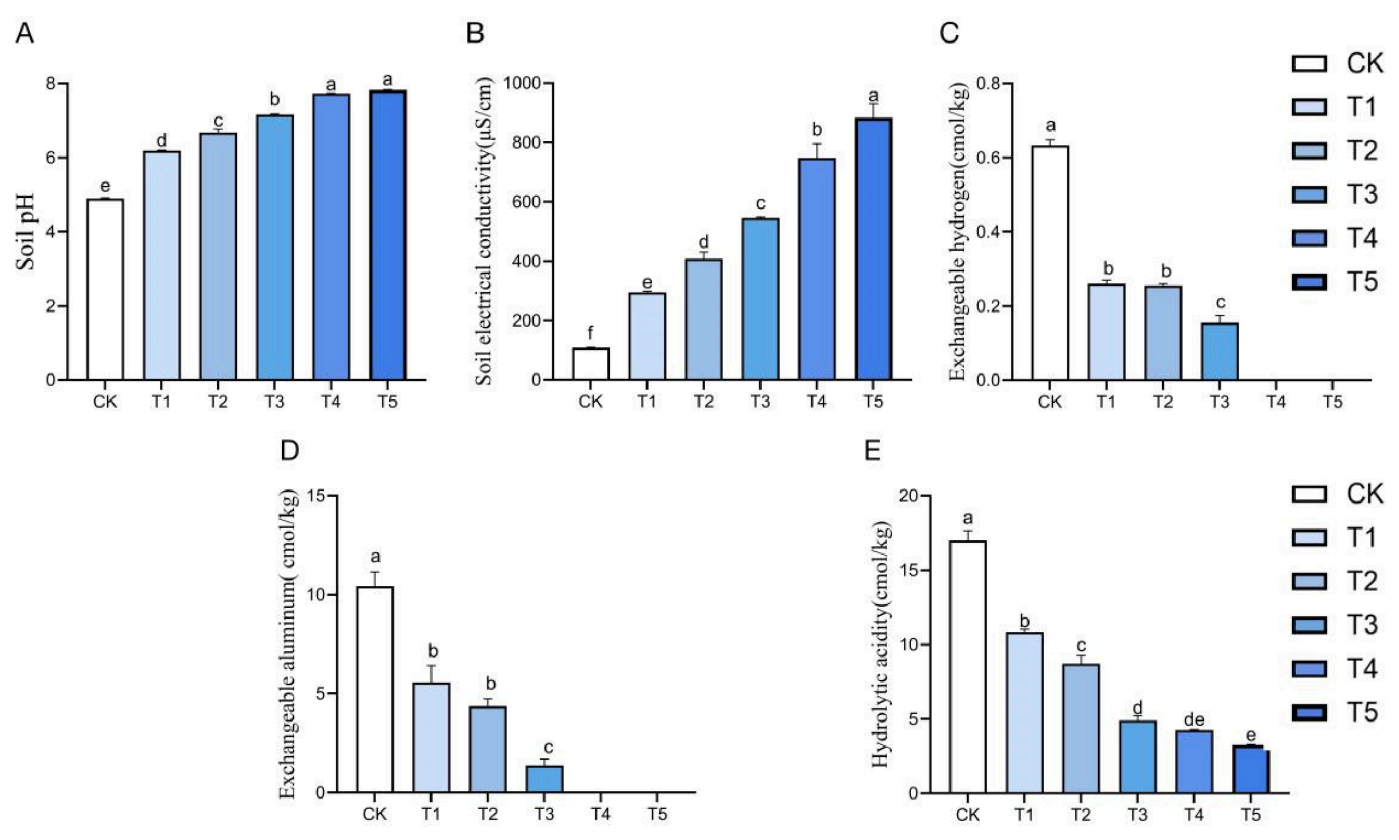
Effects of *Pseudomonas fluorescens* application at different concentrations on soil acidification-related indicators. (**A**) Soil pH; (**B**) soil electrical conductivity (EC, μS cm^−1^); (**C**) exchangeable hydrogen content (cmol kg^−1^); (**D**) exchangeable aluminum content (cmol kg^−1^); (**E**) hydrolytic acidity (cmol kg^−1^). CK: control without bacterial inoculation; T1–T5: treatments receiving increasing concentrations of *Pseudomonas fluorescens*. Values are presented as means ± standard deviation (SD) of four independent biological replicates (*n* = 4). Different lowercase letters indicate significant differences among treatments according to one-way ANOVA followed by Tukey’s multiple comparison test (*p* < 0.05).

The results regarding soil pH, EC, soil exchangeable hydrogen, aluminum, and hydrolytic acids indicate that compared with the control (CK), treatments with different concentration gradients of *Pseudomonas fluorescens* (T1–T5) all had a significant improving effect on the physical and chemical properties of acidified soil. One-way ANOVA revealed significant treatment effects on soil pH (F_(5,18)_ = 270, *p* < 0.0001, r^2^ = 0.991); soil EC (F_(5,18)_ = 297.9, *p* < 0.0001, r^2^ = 0.992); exchangeable hydrogen (F_(5,18)_ = 1304, *p* < 0.0001, r^2^ = 0.998); exchangeable aluminum (F_(5,18)_ = 340, *p* < 0.0001, r^2^ = 0.993); and hydrolytic acid (F_(5,18)_ = 548.6, *p* < 0.0001, r^2^ = 0.996). Specifically, each treatment significantly increased soil pH value and EC, while significantly reducing the contents of exchangeable hydrogen, exchangeable aluminum, and total hydrolytic acid (Figure 1). With the increase in application concentration, the degree of improvement of the above indicators related to soil acidification showed a gradually increasing trend, indicating a concentration-dependent alleviation of soil acidity-related properties. The T2 treatment turned the soil from acidic (pH < 6.5) to neutral (pH = 6.68); the T3 treatment further increased the soil pH to the alkaline range (7.16); and by the T5 treatment, the soil pH reached 7.83 (Figure 1A). At the same time, the soil EC value in each treatment from T1 to T5 increased by 171.69% to 715.87% compared with CK (Figure 1B), indicating a marked increase in the ionic strength of the soil extract. The high-concentration T4–T5 treatments reduced the contents of exchangeable hydrogen and exchangeable aluminum to below the detection limit (Figure 1C,D), and hydrolytic acidity decreased to approximately one-third to one-quarter of the CK level (Figure 1E). Collectively, these findings indicate that inoculation was associated with substantial alleviation of soil acidity, as reflected by increased pH and reduced exchangeable and hydrolytic acidity.

Meanwhile, inoculation with *Pseudomonas fluorescens* at different concentrations significantly increased the contents of exchangeable cations in acidified soil, thereby substantially altering the exchangeable base-cation pool. One-way ANOVA revealed significant treatment effects on exchangeable Ca^2+^ (F_(5,18)_ = 301.8, *p* < 0.0001, r^2^ = 0992); exchangeable Na^+^ (F_(5,18)_ = 47.36, *p* < 0.0001, r^2^ = 0.952); exchangeable K^+^ (F_(5,18)_ = 203, *p* < 0.0001, r^2^ = 0.988); and exchangeable Mg^2+^ (F_(5,18)_ = 93.12, *p* < 0.0001, r^2^ = 0.975). The contents of exchangeable Ca^2+^ and exchangeable Na^+^ increased progressively with increasing bacterial application rate: exchangeable Ca^2+^ rose from 1.28 cmol kg^−1^ in T1 to 2.39 cmol kg^−1^ in T5 (Figure S1A), while exchangeable Na^+^ increased from 4.84 cmol kg^−1^ in T1 to 6.91 cmol kg^−1^ in T5 (Figure S1B). In contrast, exchangeable K^+^, exchangeable Mg^2+^, and total exchangeable cations exhibited a unimodal trend (first increasing, then decreasing), with the highest values observed in the T3 treatment.

**Figure 2:**
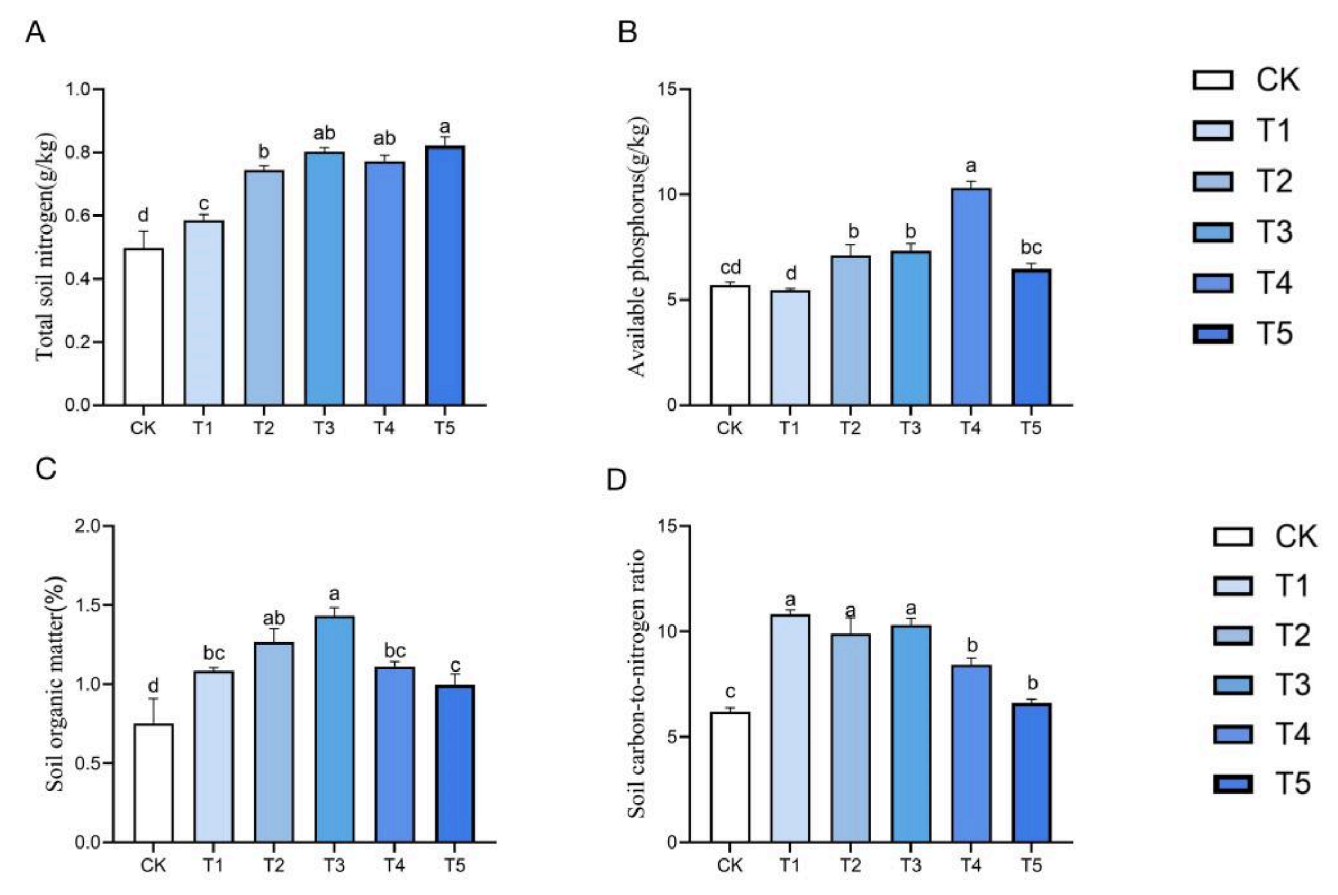
Effects of *Pseudomonas fluorescens* application at different concentrations on soil nutrient status. (**A**) Total soil nitrogen (g kg^−1^); (**B**) available phosphorus (mg kg^−1^); (**C**) soil organic matter (%); (**D**) soil carbon-to-nitrogen (C/N) ratio. CK: control without bacterial inoculation; T1–T5: treatments receiving increasing concentrations of *Pseudomonas fluorescens*. Values are presented as means ± standard deviation (SD) of four independent biological replicates (*n* = 4). Different lowercase letters indicate significant differences among treatments according to one-way ANOVA followed by Tukey’s multiple comparison test (*p* < 0.05).

Inoculation with *Pseudomonas fluorescens* significantly improved the nutrient status of acidified soil, with all indices exhibiting significant concentration-dependent differences (Figure 2). One-way ANOVA revealed significant treatment effects on TN (F_(5,18)_ = 164.1, *p* < 0.0001, r^2^ = 0.979), AP (F_(5,18)_ = 90.40, *p* < 0.0001, r^2^ = 0.974), SOM (F_(5,18)_ = 42.78, *p* < 0.0001, r^2^ = 0.947), and the C/N ratio (F_(5,18)_ = 164.5, *p* < 0.0001, r^2^ = 0.986). Overall, soil TN content increased continuously with increasing bacterial application rate, peaking at 0.82 g kg^−1^ in the T5 treatment, representing a significant—64%—increase relative to the control (Figure 2A). In contrast, AP, SOM, and the C/N ratio followed a unimodal trend (first increasing, then decreasing), showing distinct concentration-dependent response patterns among nutrient indices. Specifically, AP reached a maximum of 10.31 mg kg^−1^ in the T4 treatment, a significant 81.19% increase compared with the control (Figure 2B). SOM content peaked at 1.43% in the T3 treatment, with a 90.67% increase relative to the control—the largest improvement among all measured indices (Figure 2C). The C/N ratio was highest in the T1, T2, and T3 treatments, but declined significantly in T4 and T5 as application rate increased, decreasing by 5.16% and 24.96% relative to the control, respectively (Figure 2D). Collectively, these results demonstrate that *Pseudomonas fluorescens* effectively enhances the nutrient status of acidified soil, with each nutrient index exhibiting a distinct suitable application concentration range.

Different application rates of *Pseudomonas fluorescens* significantly affected soil acid–base buffering capacity (Figure S2). In the soil acid–base buffering curve, the slope (K) of the fitted linear regression line quantifies buffering capacity, with a smaller slope indicating a stronger ability to resist acid–base fluctuations. Overall, soil acid buffering capacity exhibited a unimodal trend (first increasing, then decreasing) with increasing bacterial application rate, with the most pronounced improvement observed in the T4 and T5 treatments. In contrast, soil alkali buffering capacity decreased continuously with rising application concentration, reaching its maximum value in the T3 treatment. Collectively, these findings demonstrate that inoculation with *Pseudomonas fluorescens* can significantly enhance soil acid buffering capacity.

### 3.2. Effects of Pseudomonas fluorescens on the Growth and Physiological Indicators of Maize at the Seedling Stage

Inoculation with *Pseudomonas fluorescens* at concentrations T1–T4 significantly promoted maize growth, nutrient accumulation, and photosynthetic performance. Notably, the T3 treatment yielded the highest plant height, thickest stem diameter, greatest aboveground and root dry weights (Figure S3), longest total root length, and largest root surface area (Figure S5). One-way ANOVA revealed significant treatment effects on plant height (F_(5,18)_ = 37.39, *p* < 0.0001, r^2^ = 0.940); root dry weights (F_(5,18)_ = 330.5, *p* < 0.0001, r^2^ = 0.993); shoot dry weight (F_(5,18)_ = 151.6, *p* < 0.0001, r^2^ = 0.984); shoot fresh weight (F_(5,18)_ = 161.2, *p* < 0.0001, r^2^ = 0.985); stem diameter (F_(5,18)_ = 113.2, *p* < 0.0001, r^2^ = 0.980); root length (F_(5,18)_ = 151.6, *p* < 0.0001, r^2^ = 0.984); root surface area (F_(5,18)_ = 85.69, *p* < 0.0001, r^2^ = 0.973); root volume (F_(5,18)_ = 288.8, *p* < 0.0001, r^2^ = 0.992). Collectively, these results show that maize growth responses differed among inoculation rates. However, when the application concentration increased to the T5 level, all maize growth and nutrient accumulation indices decreased significantly. *Pseudomonas fluorescens* application also exerted significant effects on maize seedling root growth (Figure S5). Within T1–T4, total root length and root surface area peaked in T3, increasing by 79.32% and 99.09% compared with the control, respectively. Total root length decreased slightly in T4, while root surface area continued to rise. At T5, both total root length and root surface area were significantly inhibited, decreasing by 28.26% and 1.21% relative to the control. In contrast, root volume increased across all treatments (29.06–222.61% above control), with the maximum observed in T2. Collectively, these results demonstrate that *Pseudomonas fluorescens* effectively promotes root elongation and expansion, though high concentrations suppress total root length and surface area.

Inoculation with *Pseudomonas fluorescens* at T1–T4 significantly enhanced maize photosynthetic function and gas exchange (Figure S4). One-way ANOVA revealed significant treatment effects on net photosynthetic rate (F_(5,18)_ = 384.2, *p* < 0.0001, r^2^ = 0.994), chlorophyll content (F_(5,18)_ = 327.9, *p* < 0.0001, r^2^ = 0.993), and transpiration rate (F_(5,18)_ = 186.5, *p* < 0.0001, r^2^ = 0.987). Net photosynthetic rate peaked in T4, increasing by 61.94% relative to the control (Figure S4A), while chlorophyll content was highest in T3 (47.78% increase; Figure S4B). Transpiration rate rose continuously with application rate, reaching a 98.33% increase over the control in T5 (Figure S4C). However, the promotive effects on net photosynthetic rate and chlorophyll content diminished significantly at T5, with no significant difference in photosynthetic rate from the control, and chlorophyll content increasing by only 12.45%.

For seedling-stage nutrient accumulation, T1–T4 significantly enhanced N, P, and K accumulation in maize roots, stems, and leaves (Figure S6). One-way ANOVA revealed significant treatment effects on root TN (F_(5,18)_ = 216.8, *p* < 0.0001, r^2^ = 0.989); root TP (F_(5,18)_ = 31.28, *p* < 0.0001, r^2^ = 0.930); root TP (F_(5,18)_ = 191.4, *p* < 0.0001, r^2^ = 0.988); stem TN (F_(5,18)_ = 90.57, *p* < 0.0001, r^2^ = 0.974); stem TP (F_(5,18)_ = 260, *p* < 0.0001, r^2^ = 0.991); stem TK (F_(5,18)_ = 184.5, *p* < 0.0001, r^2^ = 0.987); leaf TN (F_(5,18)_ = 74.72, *p* < 0.0001, r^2^ = 0.969); leaf TP (F_(5,18)_ = 762.1, *p* < 0.0001, r^2^ = 0.997); and leaf TK (F_(5,18)_ = 69.27, *p* < 0.0001, r^2^ = 0.976). T3 achieved the highest root TN and leaf TK, while T2 maximized leaf TN and stem TP. Notably, *Pseudomonas fluorescens* inoculation exerted an extremely significant promotive effect on root TK and leaf TP, with increases of up to 1246.67% and 1120.0% relative to the control, respectively. These findings confirm that a suitable application rate effectively improves nutrient absorption and distribution in maize seedlings, though several indices (e.g., root TN, stem TK) decreased significantly at the T5 concentration.

Pearson correlation analysis revealed significant associations between soil physicochemical properties and maize growth, as well as nutrient uptake indicators during the remediation of acidified soil by *Pseudomonas fluorescens* (Figure S7). Soil exchangeable hydrogen, exchangeable aluminum, and soil pH exhibited significant correlations with maize leaf TP, with exchangeable H^+^ and exchangeable Al^3+^ showing extremely strong negative correlations (R = −0.983, −0.974, respectively, *p* < 0.0001, *p* = 0.0010, respectively) and soil pH showing an extremely strong positive correlation (R = 0.970, *p* = 0.0013). Additionally, soil total exchangeable cations were significantly positively correlated with maize stem TN (R = 0.908, *p* = 0.0120), and soil exchangeable potassium was significantly positively correlated with stem TP (R = 0.895, *p* = 0.0160), indicating close associations between soil exchangeable cation status and N and P accumulation in maize stems.

### 3.3. Effects of Pseudomonas fluorescens on Rhizosphere Soil Microorganisms

In this study, 16S rRNA gene sequencing was performed on 24 soil samples, including 6 treatments (CK and T1–T5) with 4 biological replicates each. A total of 2,046,142 clean reads and 859.68 Mb of data were generated, with an average of 37.96 Mb and 420 bp per sample. After taxonomic annotation, 20,159 OTUs were identified, with an average of 840 OTUs per sample.

**Figure 3:**
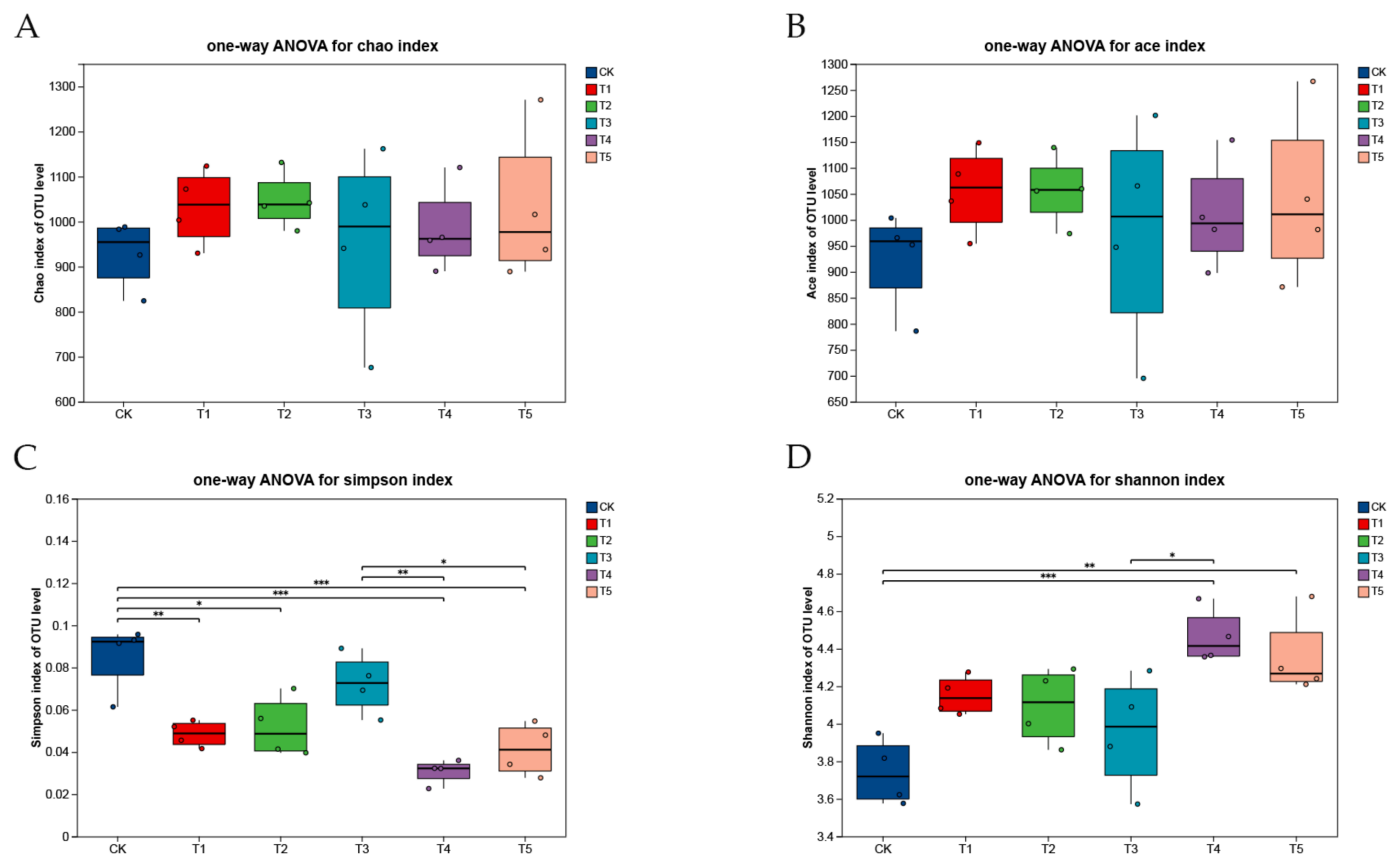
Effects of *Pseudomonas fluorescens* application at different concentrations on soil bacterial diversity indices. (**A**) Chao index; (**B**) ACE index; (**C**) Simpson index; (**D**) Shannon index. CK: untreated control; T1–T5: treatments with increasing concentrations of *Pseudomonas fluorescens*. Different lowercase letters above box plots indicate significant differences among treatments at *p* < 0.05 (one-way ANOVA). *, **, and *** represent significant differences at *p* < 0.05, *p* < 0.01, and *p* < 0.001, respectively.

**Figure 4:**
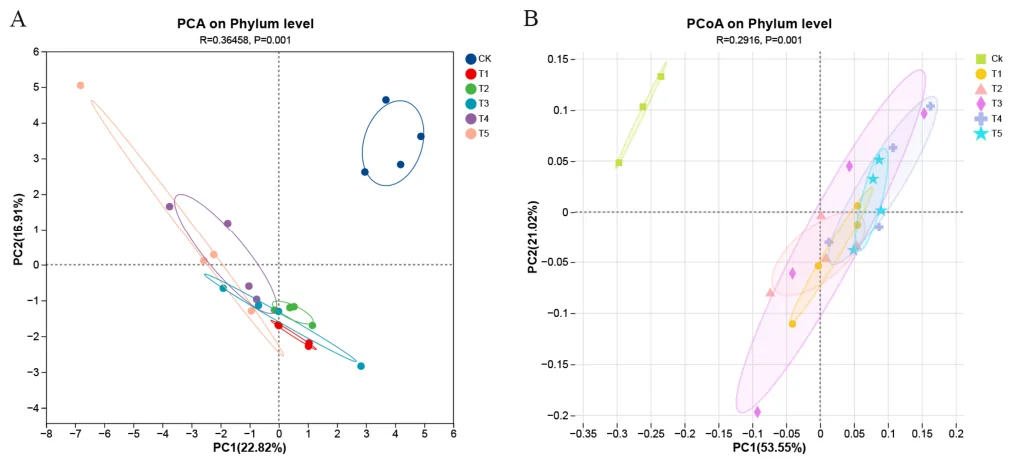
(**A**,**B**) Principal component analysis (PCA) at the phylum level and principal coordinate analysis (PCoA) based on Bray–Curtis distance at the OTU level of soil bacterial community structure under different concentrations of *Pseudomonas fluorescens* application. CK: untreated control; T1–T5: treatments with increasing concentrations of *Pseudomonas fluorescens*.

No significant differences in Chao and ACE indices (species richness) were observed across treatments (Figure 3A,B). Significantly higher Shannon index values (*p* = 0.0078) were recorded for all inoculated treatments, alongside a decreasing trend in the Simpson index (Figure 3C). The T4 treatment had the highest Shannon index (Figure 3D), while CK exhibited the highest Simpson index, followed by T3 (Figure 3D). Principal component analysis (PCA, Figure 4A) showed distinct clustering patterns of soil bacterial communities under different *Pseudomonas fluorescens* inoculation concentrations. PC1 and PC2 explained 22.82% and 16.91% of the total variation, respectively, with a cumulative contribution of 39.73%. ANOSIM analysis yielded *p* = 0.001, revealing larger variation between treatments than within-treatment replicates. Samples from CK were clearly separated from all inoculated-treatment samples along the PC1 and PC2 axes. Principal coordinate analysis (PCoA) based on Bray–Curtis distance was performed at the phylum level (Figure 4B). PC1 and PC2 explained 53.55% and 21.02% of the total community variation, respectively, with a cumulative explanation of 74.57%. CK samples were clearly separated from all inoculant treatments (T1–T5), and inoculated samples presented a gradient shift along the positive PC1 axis. The ANOSIM analysis returned R = 0.2916, *p* = 0.001. T1 and T2 shared similar community distribution. Along with the increasing inoculant dosage, T3–T5 samples further shifted towards positive PC1, and T4 and T5 exhibited high similarity in community composition. Larger within-group dispersion was observed for T5.

**Figure 5:**
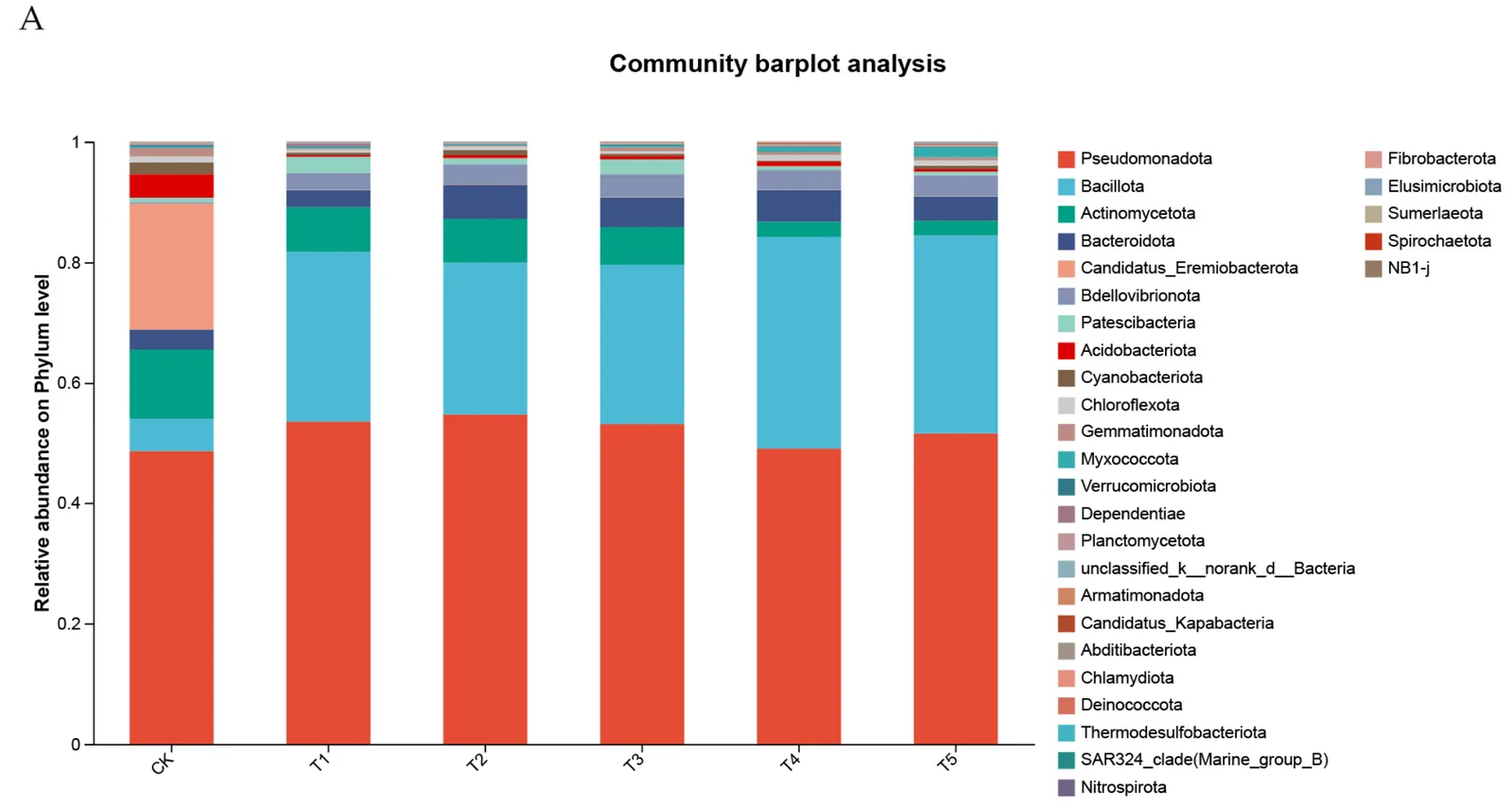

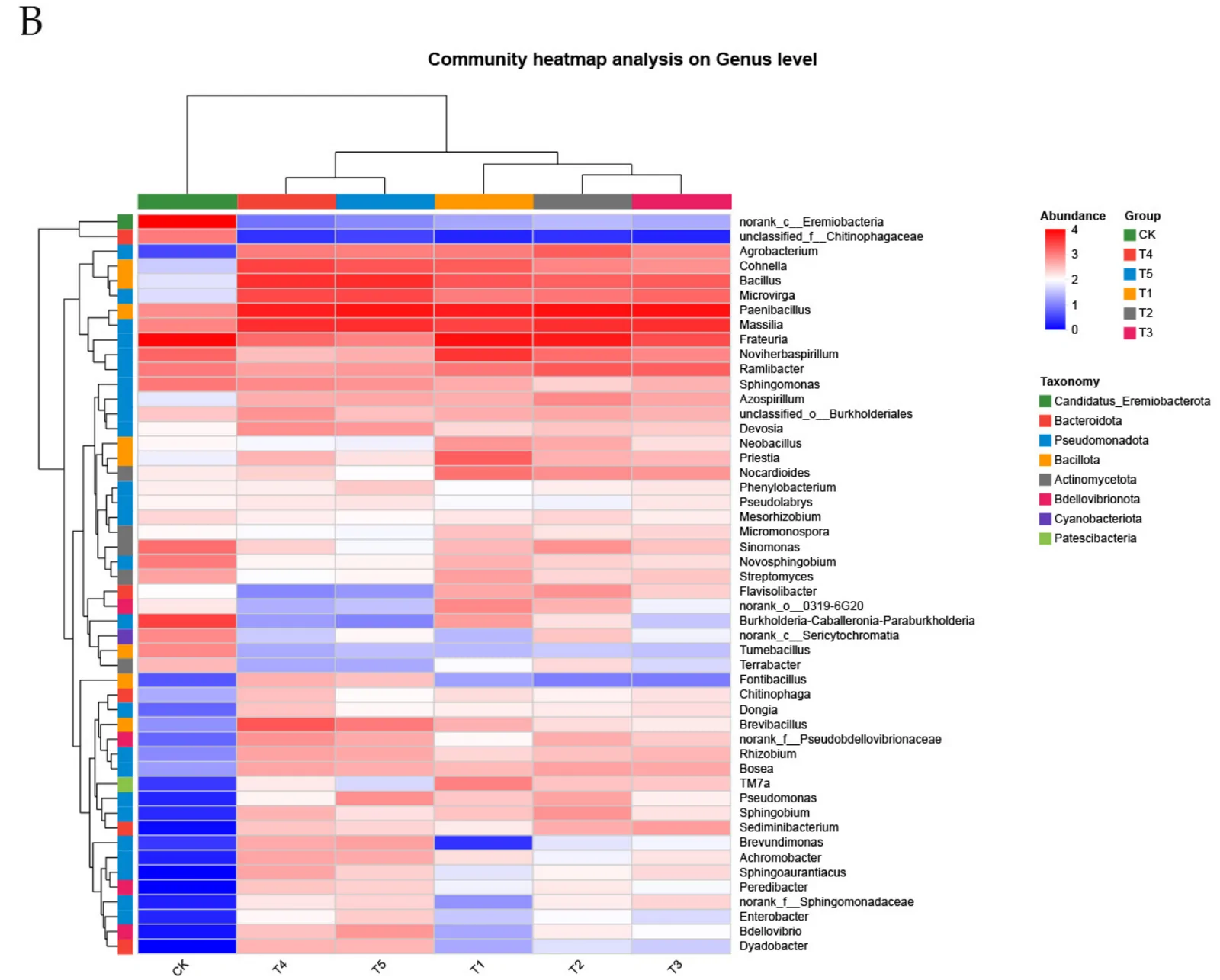
Effects of *Pseudomonas fluorescens* application at different concentrations on soil bacterial community composition. (**A**) Relative abundance (%) of bacterial communities at the phylum level; (**B**) genus-level bacterial community heatmap, with color intensity representing relative abundance. Microbial community analysis was based on four independent biological replicates per treatment (*n* = 4).

At the phylum and genus levels, community composition differed markedly between CK and treated soils (Figure 5). Pseudomonas (Proteobacteria) was the most dominant genus in all inoculated treatments, reaching a maximum relative abundance of 54.73% in T2. *Bacillus* (Firmicutes) was the subdominant genus (25.20–35.22%) across T1–T5, whereas Candidatus_Eremiobacterota was dominant in CK. The relative abundance of Actinomycetota decreased under inoculation, being highest (11.48%) in the control. Genus-level heatmap analysis further revealed that inoculation with *Pseudomonas fluorescens* significantly increased the relative abundance of several bacterial genera, including *Fontibacillus*, Chitinophaga, *Rhizobium*, Bosea, and Bdellovibrio, while significantly suppressing norank_c__Eremiobacteria and unclassified Chitinophagaceae.

Microbial ecological-strategy analysis presented varying proportions of persistent, intermittent, and transient taxa along the inoculant concentration gradient (Figure S8). Higher proportions of persistent and intermittent taxa and lower proportions of transient taxa were found in T1–T4. The T2 treatment had the highest proportion of persistent taxa (92.06%) and the lowest proportion of transient taxa (3.4%). Lower proportions of persistent taxa and higher proportions of transient and intermittent taxa were observed in T5. Genus-level Venn diagram analysis (Figure S7) showed that T3 contained the largest number of unique OTUs (191). A total of 350 core OTUs were shared by all treatments.

**Figure 6:**
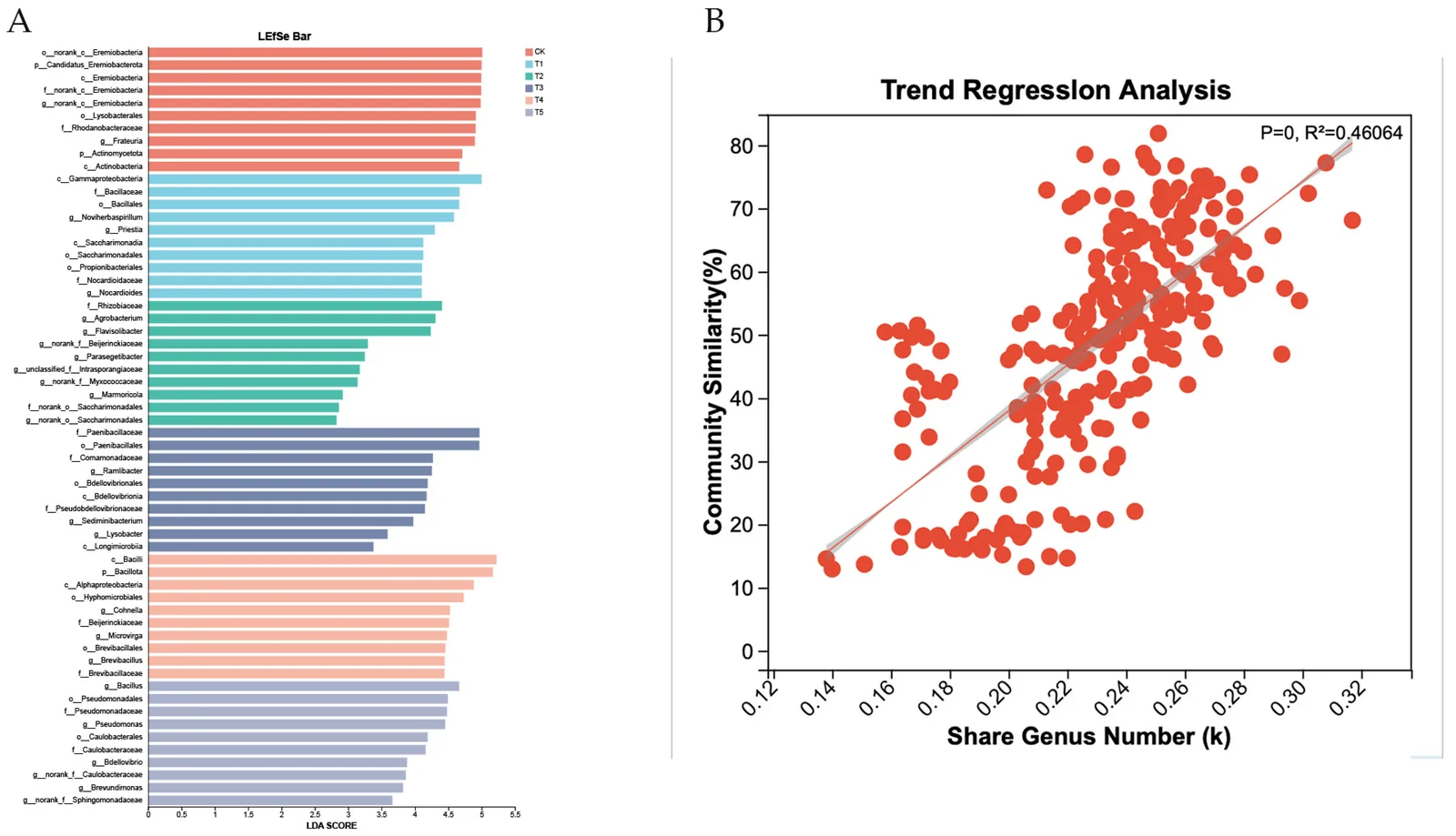
(**A**) LEfSe analysis of soil bacterial communities under different concentrations of *Pseudomonas fluorescens* application. (**B**) Trend regression analysis between the number of shared genera and community similarity.

LEfSe analysis (LDA > 3.0, Figure 6A) detected differential biomarker taxa for each treatment. Enrichment of Proteobacteria and Firmicutes occurred in all inoculated treatments. T1 was characterized by Gammaproteobacteria, *Bacillaceae*, and *Bacillales*; T2 by Rhizobiaceae, *Agrobacterium*, and *Flavisolibacter*; T3 by Paenibacillaceae, Paenibacillales, and Comamonadaceae; T4 by Bacilli, Firmicutes, and Alphaproteobacteria; T5 by *Bacillus*, Pseudomonadales, and Pseudomonadaceae.

Regression analysis (Figure 6B) showed a highly significant positive correlation between the number of shared genera and community similarity (*p* = 0, r^2^ = 0.461). Approximately 46% of the variance in community similarity could be explained by the number of shared genera.

## 4. Discussion

This study demonstrated that *Pseudomonas fluorescens* inoculation exerted concentration-dependent effects on acidified soil properties, maize seedling growth, and rhizosphere microbial communities. Moderate inoculation rates showed the most favorable overall response patterns, indicating that microbial-mediated soil amelioration is governed by a suitable inoculation threshold rather than a simple dose-dependent enhancement. Previous studies have demonstrated that the mechanisms by which *Pseudomonas fluorescens* ameliorates acidified soil and regulates soil physicochemical properties may involve both direct physicochemical effects and indirect microbial regulation [31,32]. Its direct effects have been proposed to involve the metabolism and surface characteristics of bacterial cells [33]. The strain may produce active substances such as organic acids and exopolysaccharides [34], which have been reported to promote mineral weathering and the release of base cations such as calcium, magnesium, and potassium [35]. Meanwhile, organic anions on the cell surface have been reported to bind H^+^ in soil, potentially contributing to the neutralization of soil acidity and reductions in exchangeable and hydrolytic acidity. Within an appropriate pH range, these processes may contribute to changes in exchangeable base cation composition, nutrient availability, and soil acid-buffering capacity [36]. Indirect effects may involve reshaping the rhizosphere microbial community [37]. Previous studies have reported that inoculation with *Pseudomonas fluorescens* may affect soil microbial community evenness and stability [38]. It may favor the enrichment of beneficial groups such as Proteobacteria and Bacillota, as well as typical plant growth-promoting bacteria, including *Bacillus* and *Paenibacillus* [39]. By regulating the rhizosphere microecology and facilitating soil nutrient cycling and organic matter accumulation, the strain may indirectly contribute to the improvement of soil physicochemical conditions and the alleviation of soil acidification [37]. In terms of soil physicochemical properties, *Pseudomonas fluorescens* significantly increased soil pH, reduced exchangeable and hydrolytic acidity, and elevated exchangeable base ion contents. These measured responses are consistent with previous reports suggesting that microbial metabolites and cell-surface interactions may promote mineral weathering, base-cation release, and H^+^ binding [31,40]. However, organic acid production, H^+^ adsorption, and mineral weathering were not directly quantified in the present study; therefore, these processes should be regarded as potential mechanisms rather than directly demonstrated pathways. Previous studies have also reported increased SOM following *Pseudomonas fluorescens* inoculation [41].

However, the increase in soil pH should not be interpreted as a continuous improvement with increasing inoculant application. Although neutralization of soil acidity can alleviate H^+^ and Al^3+^ toxicity and improve nutrient availability in strongly acidic soils, excessive alkalization may also adversely affect soil nutrient availability [42]. Moderate inoculation of *Pseudomonas fluorescens* effectively elevates soil pH and alleviates soil acidification, whereas excessive inoculum dosage can further shift soil conditions from acidic to weakly alkaline. Under such conditions, the availability of some nutrients, particularly P and micronutrients such as Fe, Mn, and Zn, may decrease because of reduced solubility or precipitation [43]. Therefore, the strongest increase in soil pH does not necessarily represent the suitable soil condition, and the beneficial effect of *Pseudomonas fluorescens* should be considered within an appropriate application range. This may also partly explain why the highest inoculation rate (T5) improved several soil acidity-related indicators but inhibited maize growth. High inoculation rates were accompanied by a marked increase in soil EC, which may increase the osmotic pressure of the soil solution and impose osmotic stress on maize roots, potentially impairing photosynthetic performance and nutrient acquisition [44,45]. Because increasing inoculation rates also involved increasing amounts of the commercial formulation, the pronounced EC increase may reflect the combined contributions of bacterial inoculation and soluble components associated with the formulation. These findings indicate that the soil amelioration and growth-promoting effects of *Pseudomonas fluorescens* exhibit an obvious dose threshold. Excessive application may disturb the physicochemical conditions of the rhizosphere microenvironment and generate negative stress effects that inhibit maize seedling growth. Therefore, future field applications of *Pseudomonas fluorescens* should consider the inoculation rate carefully to balance soil acidity alleviation with the potential adverse effects associated with excessive application, such as elevated EC and osmotic stress.

The present study extends previous understanding by demonstrating that the soil amelioration effect of *Pseudomonas fluorescens* is not simply proportional to inoculation rate, but follows a concentration-dependent pattern. At low to moderate inoculation rates (T1–T4), the increasing abundance of introduced bacteria likely enhanced their interactions with soil minerals and indigenous microorganisms, thereby promoting acidity alleviation, nutrient transformation, and rhizosphere improvement (Figure 1, Figure 2 and Figure 3). However, when the inoculation rate exceeded a suitable threshold, further increases in bacterial input did not translate into additional ecological benefits. Instead, excessive inoculation altered the rhizosphere chemical environment, as indicated by the continued increase in soil pH and EC, which may have resulted in excessive alkalization and osmotic stress. Therefore, our results reveal that the effectiveness of microbial amelioration depends not only on the functional capacity of the inoculated strain but also on maintaining an appropriate inoculation intensity that balances microbial activity and rhizosphere environmental stability [28].

Additionally, although all treatments received the same amount of urea, soil TN increased progressively with increasing inoculant application rate. This response may be partly related to the increasing amount of commercial microbial formulation added across treatments (10–60 g pot^–1^), which introduced different amounts of microbial biomass and carrier materials and may therefore have contributed additional N to the soil [46]. In addition, inoculation with *Pseudomonas fluorescens* may have altered rhizosphere microbial activity, plant N uptake, and N retention or loss processes, thereby affecting the amount of N remaining in the soil [47]. The contrasting patterns of TN and SOM are not necessarily inconsistent, because these pools are regulated by different biogeochemical processes [45]; in this study, SOM peaked at T3 and subsequently declined, whereas TN continued to increase, resulting in a marked decrease in the soil C/N ratio under the higher inoculation rates. Therefore, the increase in TN likely reflects the combined effects of inoculant-associated N input and altered soil N cycling rather than a direct proportional increase in SOM.

Regarding crop responses, an appropriate concentration of *Pseudomonas fluorescens* significantly promoted maize growth, photosynthetic performance, and root development. This may be attributed to its ability to secrete phytohormones (e.g., indole-3-acetic acid, IAA) to stimulate root elongation and branching [48], expanding the root absorption area [49] and enhancing water and nutrient acquisition [50]. Previous studies have also indicated that *Pseudomonas fluorescens* regulates rhizosphere nutrient availability via organic acids, extracellular enzymes, and siderophores [51], with siderophores and organic acids improving the availability of key nutrients (e.g., Fe, P) to enhance plant nutrition. In this study, T1–T4 treatments promoted maize growth, nutrient accumulation, and photosynthesis, with T3 yielding the highest plant height, stem diameter, aboveground/root dry weights, total root length, and root surface area, confirming that a suitable inoculation rate enhances maize seedling morphogenesis and dry matter accumulation. Conversely, T5 significantly inhibited these indices. It should be noted that the identification of T3 and T4 as favorable treatments was based on the overall response pattern rather than a single indicator. Different biological processes exhibited distinct concentration-dependent responses; for example, root morphology and several nutrient-related traits showed the strongest responses under T3, whereas some photosynthetic and microbial diversity-related indicators showed higher responses under T4. Therefore, T3 and T4 represent a favorable inoculation range rather than a universal optimum for all individual variables.

Regarding microbial community structure, Proteobacteria and Bacillota were significantly enriched in inoculated soils. This enrichment reflects both direct inoculation effects and the proliferation of copiotrophic r-strategist Proteobacteria under improved nutrient conditions [52,53]. The enrichment of Bacillota may reflect altered resource availability and microbial interactions following inoculation, although these mechanisms were not directly examined in the present study [54]. The increased proportion of persistent taxa indicates a greater contribution of frequently detected taxa to the bacterial assemblage under inoculation. However, because samples were collected at a single time point, this pattern should not be interpreted as direct evidence of temporal community stability [55].

In this study, the relative abundance of Actinomycetota decreased during soil remediation, which was inconsistent with the previously reported increase in Actinomycetota abundance during soil improvement [56]. This discrepancy can be attributed to the ecological traits of Actinomycetota, which exhibit a stronger competitive advantage under oligotrophic and stressful soil conditions. Inoculation with *Pseudomonas fluorescens* improved soil nutrient status and promoted the proliferation of copiotrophic microorganisms such as Proteobacteria, which may have reduced the relative competitive advantage of oligotrophic Actinomycetota.

Although the basic soil amelioration mechanisms observed in this study are consistent with previous findings, the present work provides clear innovative insights. Most previous studies have emphasized the beneficial effects of microbial inoculation under selected application rates, whereas the potential threshold responses associated with increasing inoculation intensity remain poorly understood. This study systematically clarifies the dose-dependent effects of *Pseudomonas fluorescens* in ameliorating acidified red soil. Low and moderate inoculation dosages effectively alleviate soil acidification, optimize the rhizosphere microenvironment, and promote maize growth. In contrast, high inoculation dosages further improve soil acidification indicators but disrupt rhizosphere microecological homeostasis, causing soil over-alkalization, increased EC, and nutrient imbalance, thereby inhibiting maize seedling growth. These findings supplement the dose-response mechanism of *Pseudomonas fluorescens* application and provide a preliminary basis for evaluating appropriate inoculation ranges in future field applications.

This study also has several limitations. All experiments were conducted under controlled pot conditions with relatively stable environmental settings, whereas field soils are subject to greater spatial heterogeneity and fluctuations in temperature, rainfall, moisture, nutrient supply, and interactions with native microbial communities. Root growth, inoculant dispersal, and bacterial persistence may therefore differ substantially between confined pot systems and field environments, which could influence both the magnitude and persistence of the observed amelioration effects. Accordingly, the suitable inoculation range and threshold responses identified here should be regarded as pot-scale evidence and require further validation across field conditions, soil types, and cropping systems. In addition, the present study focused exclusively on rhizosphere bacterial communities and did not account for fungi or other microbial groups, limiting a more comprehensive interpretation of rhizosphere microecological responses. The use of 16S rRNA gene metabarcoding further restricts functional inference because this approach primarily resolves taxonomic composition rather than functional genes or metabolic pathways. Future studies integrating field experiments, broader microbial profiling, shotgun metagenomics, and functional assays would provide a more complete understanding of strain–soil–plant interactions.

Moreover, because the commercial inoculant was applied as a complete formulation and a carrier-only control was not included, the effects of *Pseudomonas fluorescens* cannot be fully separated from those of the carrier materials, particularly at higher application rates. Some changes in soil physicochemical properties may therefore reflect the combined effects of bacterial inoculation and formulation components. Future experiments incorporating equivalent carrier-only controls will be necessary to distinguish these contributions more precisely. Overall, the present pot experiment demonstrates concentration-dependent effects of *Pseudomonas fluorescens* inoculation on acidified soil properties, maize seedling growth, and rhizosphere bacterial communities. The identified application range provides a preliminary basis for microbial amelioration of acidified farmland, although its agronomic effectiveness and long-term stability remain to be verified under field conditions.

## 5. Conclusions

In summary, this study indicated that *Pseudomonas fluorescens* effectively ameliorates acidified soil and promotes maize seedling growth. All inoculation treatments significantly increased soil pH, decreased exchangeable acidity, and improved exchangeable base cation status to alleviate soil acidification. However, the highest inoculation rate (T5) reduced several nutrient-related parameters, including available phosphorus, soil organic matter and the C/N ratio, and induced rhizosphere stress that inhibited maize seedling growth. With respect to plant performance, T3 and T4 (1.1 × 10^10^ CFU pot^−1^ and 2.2 × 10^10^ CFU pot^−1^) markedly promoted maize growth, photosynthesis, root development, and N/P/K uptake, whereas the highest concentration (3.3 × 10^10^ CFU pot^−1^) showed inhibitory effects, indicating a clear concentration-dependent response. Microbial community analysis further revealed that *Pseudomonas fluorescens* inoculation altered rhizosphere bacterial community structure and diversity and enriched beneficial groups, including Proteobacteria and Bacillota, as well as plant growth-promoting genera such as *Bacillus*, *Pseudomonas*, and *Paenibacillus*. Overall, T3 and T4 represented the most favorable application range based on the overall response patterns of soil amelioration, maize growth, and rhizosphere bacterial community characteristics, rather than a universal optimum for all individual variables. These findings provide pot-scale evidence for the use of *Pseudomonas fluorescens* in acidified soil amelioration, while its effects on full-season crop performance, grain yield and quality, and long-term soil properties require further field evaluation.

## Data Availability

The raw 16S rRNA gene sequencing data generated in this study have been deposited in the NCBI Sequence Read Archive (SRA) under BioProject accession number PRJNA1474281 and SRA submission ID SUB16228186. The original contributions presented in this study are included in the article/Supplementary Material. Further inquiries can be directed to the corresponding author.

## Supplementary Materials

The following supporting information can be downloaded at https://www.mdpi.com/article/10.3390/plants15172679/s1. Figure S1. Effects of *Pseudomonas fluorescens* application at different concentrations on soil exchangeable cations contents. (A) Exchangeable calcium; (B) Exchangeable sodium; (C) Exchangeable potassium; (D) Exchangeable magnesium; (E) Total exchangeable bases. CK: untreated control; T1–T5: treatments with increasing concentrations of *Pseudomonas fluorescens*. Different lowercase letters above bars indicate significant differences among treatments at *p* < 0.05 (Tukey’s test). Values are presented as means ± standard deviation (SD); Figure S2. Soil acid-base buffering curves for different treatments. The x-axis represents measured soil pH, and the y-axis indicates the volume of acid/base added (negative values for acid addition, positive values for base addition). CK: untreated control; T1–T5: treatments with increasing concentrations of *Pseudomonas fluorescens*. The slope of the fitted linear segment in the curve reflects soil buffering capacity, with a smaller slope indicating a stronger buffering ability; Figure S3. Effects of *Pseudomonas fluorescens* application at different concentrations on maize seedling growth. (A) Plant height; (B) Stem diameter; (C) Shoot dry weight; (D) Root dry weight; (E) Shoot fresh weight; CK: untreated control; T1–T5: treatments with increasing concentrations of *Pseudomonas fluorescens*. Different lowercase letters above bars indicate significant differences among treatments at *p* < 0.05 (Tukey’s test). Values are presented as means ± standard deviation (SD); Figure S4. Effects of *Pseudomonas fluorescens* application at different concentrations on photosynthetic parameters in maize seedlings. (A) Net photosynthetic rate; (B) Chlorophyll content (SPAD value); (C) Transpiration rate. CK: untreated control; T1–T5: treatments with increasing concentrations of *Pseudomonas fluorescens*. Different lowercase letters above bars indicate significant differences among treatments at *p* < 0.05 (Tukey’s test). Values are presented as means ± standard deviation (SD); Figure S5. Effects of *Pseudomonas fluorescens* application at different concentrations on root morphological parameters in maize seedlings. (A) Total root length; (B) Root surface area; (C) Root volume. CK: untreated control; T1–T5: treatments with increasing concentrations of *Pseudomonas fluorescens*. Different lowercase letters above bars indicate significant differences among treatments at *p* < 0.05 (Tukey’s test). Values are presented as means ± standard deviation (SD); Figure S6. Effects of *Pseudomonas fluorescens* application at different concentrations on nutrient accumulation in maize seedlings. (A) Root total nitrogen; (B) Root total phosphorus; (C) Root total potassium; (D) Stem total nitrogen; (E) Stem total phosphorus; (F) Stem total potassium; (G) Leaf total nitrogen; (H) Leaf total phosphorus; (I) Leaf total potassium. CK: untreated control; T1–T5: treatments with increasing concentrations of *Pseudomonas fluorescens*. Different lowercase letters above bars indicate significant differences among treatments at *p* < 0.05 (Tukey’s test). Values are presented as means ± standard deviation (SD); Figure S7. Pearson correlation heatmap of soil physicochemical properties and maize seedling growth and nutrient indices. The color gradient represents the correlation coefficient (r), with red indicating a positive correlation and blue indicating a negative correlation. The values in the grid represent the correlation coefficients between two indices; Figure S8. Effects of *Pseudomonas fluorescens* application at different concentrations on soil bacterial community stability and OTU distribution. (A) Proportions of persistent, intermittent, and transient taxa in the soil bacterial community, presented by both relative abundance and number of species. (B) Venn diagram showing the shared and unique OTUs among treatments, with numbers indicating the count of unique OTUs in each group and the core OTUs shared by all treatments. CK: untreated control; T1–T5: treatments with increasing concentrations of *Pseudomonas fluorescens*.

## Abbreviations

The following abbreviations are used in this manuscript:

IAA	Indole-3-acetic acid
VOCs	Volatile organic compounds
2,4-DAPG	2,4-diacetylphloroglucinol
PCA	Phenazine-1-carboxylic acid
PCoA	Principal coordinate analysis
