# Supplementary material for: Amelioration Effects of Pseudomonas fluorescens on Acidified Soil and Subsequent Promotion of Maize Growth
Source: Plants (Basel). 2026 Aug 31;15(17):2679. doi: 10.3390/plants15172679 (PMC13567574; doi:10.3390/plants15172679)
Supplement: Supplementary file 1 [file plants-15-02679-s001.zip › plants-4436181-supplementary.pdf]

Amelioration Effects of *Pseudomonas fluorescens* on Acidified Soil and Subsequent Promotion the Growth of Maize

Tingyi Wang, Ze Yu, Meilan Chen, Lansheng Deng *

College of Natural Resources and Environment, South China Agricultural University, Guangzhou 510640, China

* Correspondence: lshdeng@scau.edu.cn; Tel.:+86-13711212595

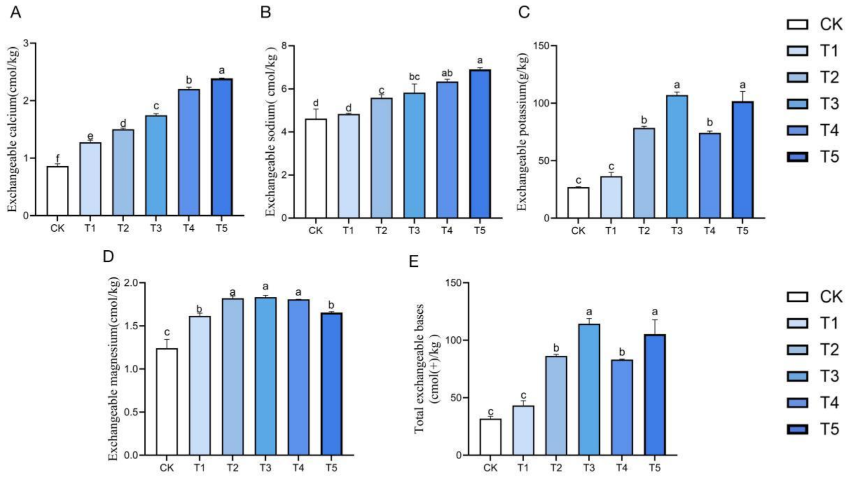

Figure S1. Effects of *Pseudomonas fluorescens* application at different concentrations on soil exchangeable cations contents. (A) Exchangeable calcium; (B) Exchangeable sodium; (C) Exchangeable potassium; (D) Exchangeable magnesium; (E) Total exchangeable bases. CK: untreated control; T1–T5: treatments with increasing concentrations of *Pseudomonas fluorescens*. Different lowercase letters above bars indicate significant differences among treatments at $p < 0.05$ (Tukey's test). Values are presented as means ± standard deviation (SD).

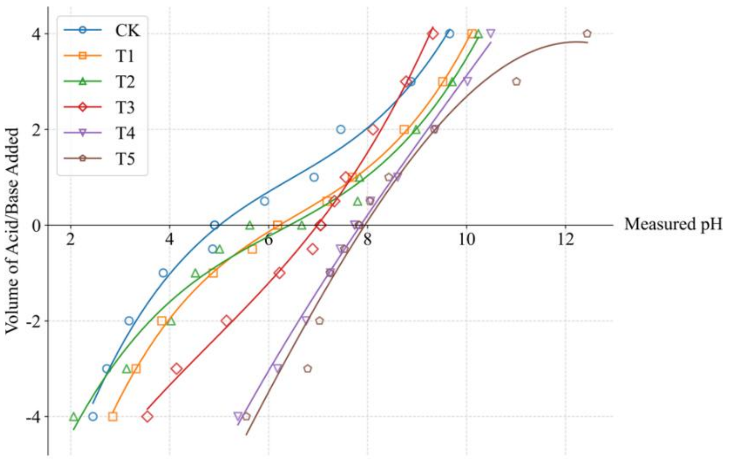

Figure S2. Soil acid-base buffering curves for different treatments. The x-axis represents measured soil pH, and the y-axis indicates the volume of acid/base added (negative values for acid addition, positive values for base addition). CK: untreated control; T1–T5: treatments with increasing concentrations of *Pseudomonas fluorescens*. The slope of the fitted linear segment in the curve reflects soil buffering capacity, with a smaller slope indicating a stronger buffering ability.

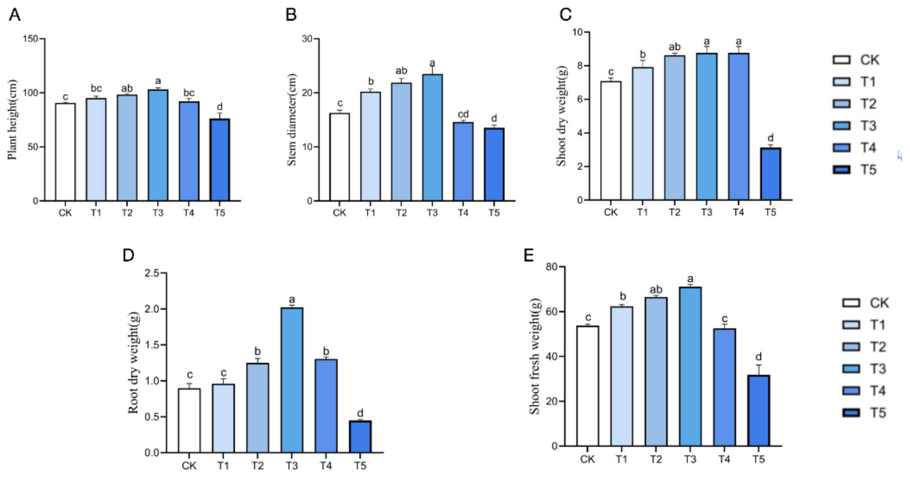

Figure S3. Effects of *Pseudomonas fluorescens* application at different concentrations on maize seedling growth. (A) Plant height; (B) Stem diameter; (C) Shoot dry weight; (D) Root dry weight; (E) Shoot fresh weight; CK: untreated control; T1–T5: treatments with increasing concentrations of *Pseudomonas fluorescens*. Different lowercase letters above bars indicate significant differences among treatments at $p < 0.05$ (Tukey's test). Values are presented as means ± standard deviation (SD).

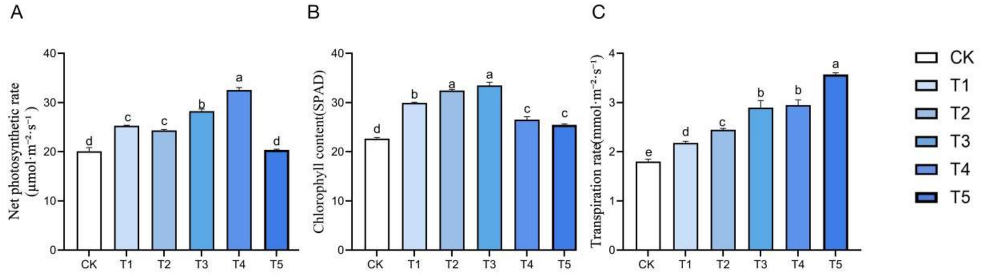

Figure S4. Effects of *Pseudomonas fluorescens* application at different concentrations on photosynthetic parameters in maize seedlings. (A) Net photosynthetic rate; (B) Chlorophyll content (SPAD value); (C) Transpiration rate. CK: untreated control; T1–T5: treatments with increasing concentrations of *Pseudomonas fluorescens*. Different lowercase letters above bars indicate significant differences among treatments at $p < 0.05$ (Tukey's test). Values are presented as means ± standard deviation (SD).

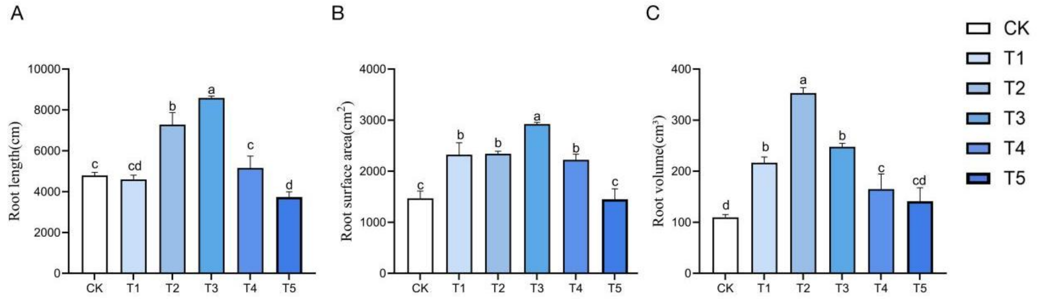

Figure S5. Effects of *Pseudomonas fluorescens* application at different concentrations on root morphological parameters in maize seedlings. (A) Total root length; (B) Root surface area; (C) Root volume. CK: untreated control; T1–T5: treatments with increasing concentrations of *Pseudomonas fluorescens*. Different lowercase letters above bars indicate significant differences among treatments at $p < 0.05$ (Tukey's test). Values are presented as means ± standard deviation (SD).

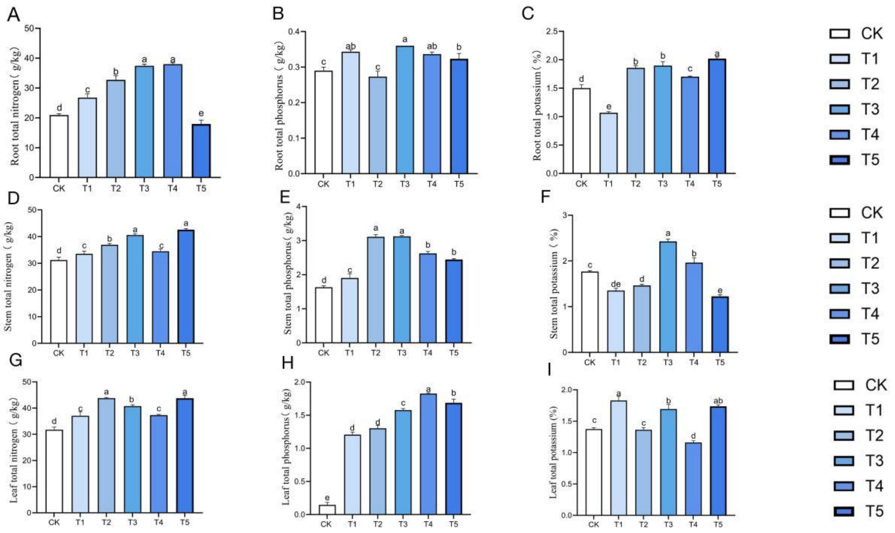

Figure S6. Effects of *Pseudomonas fluorescens* application at different concentrations on nutrient accumulation in maize seedlings. (A) Root total nitrogen; (B) Root total phosphorus; (C) Root total potassium; (D) Stem total nitrogen; (E) Stem total phosphorus; (F) Stem total potassium; (G) Leaf total nitrogen; (H) Leaf total phosphorus; (I) Leaf total potassium. CK: untreated control; T1–T5: treatments with increasing concentrations of *Pseudomonas fluorescens*. Different lowercase letters above bars indicate significant differences among treatments at $p < 0.05$ (Tukey’s test). Values are presented as means ± standard deviation (SD).

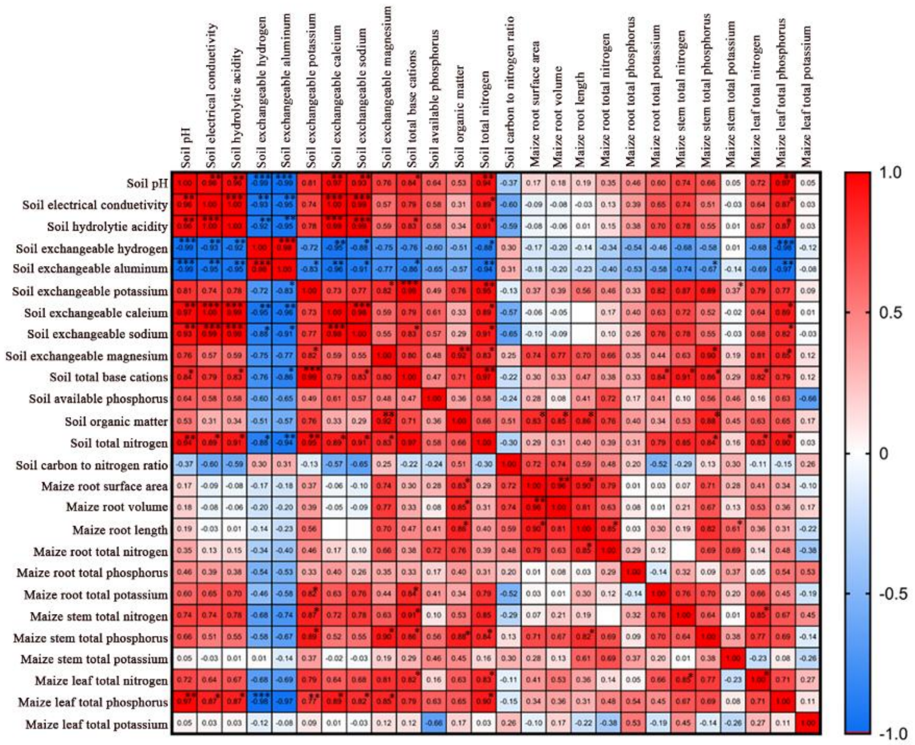

Figure S7. Pearson correlation heatmap of soil physicochemical properties and maize seedling growth and nutrient indices. The color gradient represents the correlation coefficient (r), with red indicating a positive correlation and blue indicating a negative correlation. The values in the grid represent the correlation coefficients between two indices.

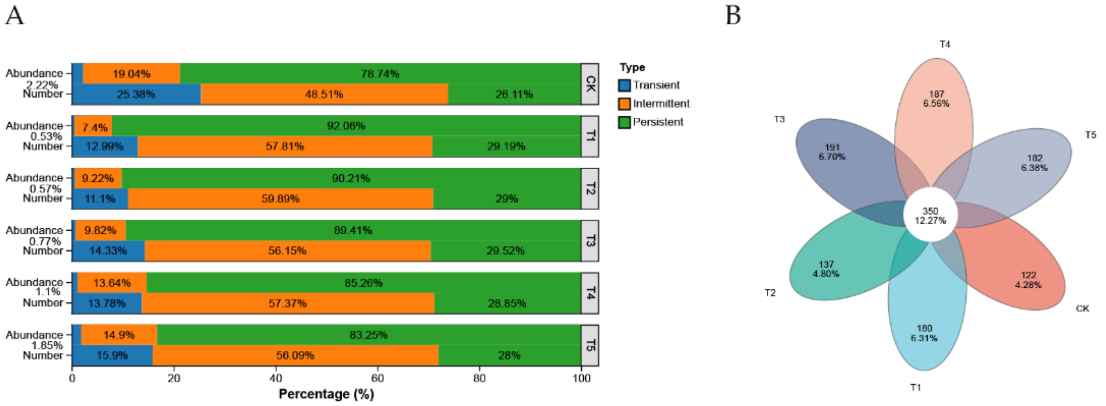

Figure S8. Effects of *Pseudomonas fluorescens* application at different concentrations on soil bacterial community stability and OTU distribution. (A) Proportions of persistent, intermittent, and transient taxa in the soil bacterial community, presented by both relative abundance and number of species. (B) Venn diagram showing the shared and unique OTUs among treatments, with numbers indicating the count of unique OTUs in each group and the core OTUs shared by all treatments. CK: untreated control; T1–T5: treatments with increasing concentrations of *Pseudomonas fluorescens*.
